# The Association between Hypoxia-Inducible Factor-1 α Gene C1772T Polymorphism and Cancer Risk: A Meta-Analysis of 37 Case-Control Studies

**DOI:** 10.1371/journal.pone.0083441

**Published:** 2013-12-18

**Authors:** Pengfei He, Qi Han, Jiajia Liu, Dongjuan Liu, Xin Zhao, Ting Hu, Lu Jiang, Hongxia Dan, Xin Zeng, Jing Li, Jiayi Wang, Qianming Chen

**Affiliations:** 1 State Key Laboratory of Oral Diseases, West China College of Stomatology, Sichuan University, Chengdu, China; 2 Department of Oral Radiology, West China Hospital of Stomatology, Sichuan University, Chengdu, China; Sapporo Medical University, Japan

## Abstract

**Background:**

The possible association between HIF-1α C1772T polymorphism and cancer risk has been studied extensively. However, the results were controversial. In order to get a more precise conclusion of this association, a meta-analysis was performed.

**Methods:**

A total of 10186 cases and 10926 controls in 37 case-control studies were included in this meta-analysis. Allele and genotypic differences between cases and controls were evaluated. Subgroup analysis by cancer site, ethnicity, source of controls and gender was performed.

**Results:**

The T allele of HIF-1α gene C1772T was significantly associated with increased cancer risk in three genetic models: TT+CT vs.CC (dominant model OR=1.23, 95%CI=1.03-1.47), TT vs. CT+CC (recessive model OR=2.51, 95%CI=1.54-4.09), TT vs. CC (homozygote comparison OR=2.02, 95%CI=1.21-3.39).In subgroup analysis, the frequency of the T variant was found to be significantly increased in cervical cancer, pancreatic cancer, head and neck cancer, renal cell carcinoma, Asian and female subgroups.

**Conclusions:**

Our meta-analysis suggests that the substitution of C allele with T at HIF-1α gene C1772T polymorphism is a risk factor of cancer, especially for cervical, head and neck cancer, pancreatic cancer and renal cell carcinoma. It is also a risk factor of cancer in Asian group as well as in female group.

## Introduction

Cancer is a multifactor disease which results from complex mutual effect between environmental and genetic factors. It has become one of the most challenging health issues today [1]. Hypoxia of cancer tissue is a hallmark of solid cancer. A hypoxic microenvironment initiates multiple cellular responses, such as proliferation and angiogenesis, resulting in the development and progression of cancer [2].

Hypoxia-inducible factor-1 (HIF-1) is a key transcription factor that regulates cellular reaction to hypoxia. It is over expressed in most solid tumors in response to low oxygen concentrations [3]. HIF-1 is a heterodimeric transcription factor consisting of two subunits, HIF-1α and HIF-1β. HIF-1α is the oxygen-regulating factor that determines HIF-1 activity. HIF-1α has the ability to induce the expression of genes whose products contribute to metabolic reprogramming, angiogenesis and metastasis. High HIF-1α levels have been associated with poor prognosis in most cancers [4–8], including epidermal carcinogenesis [9,10].

There is an important single nucleotide polymorphism (SNP) of human HIF-1α gene at 1772, the wild type cytosine (C) allele is replaced by a thymine (T) allele (named C1772T or P582S or rs11549465). This replacement leads to an amino acid substitution of proline with serine. The role of this polymorphism in various types of cancer has been widely investigated. It was found that C1772T status was associated with susceptibility to various cancer types. It was also suggested that genotypes with T allele (TT or CT) had significantly higher transcription activities of HIF-1α gene compared with wild CC genotype [3,11]. Moreover, this polymorphism may also be associated with lymph node or distal metastasis [12–14].

The possible association between HIF-1α C1772T and cancer risk has been investigated in several studies, but the results were inconclusive or even contradictory [15-39]. Although a meta-analysis on HIF-1α polymorphisms and cancer risk was conducted by Tongfeng Zhao et al [40] in 2009, it had several limitations. The sample size of this study was relatively limited (4131 cancer cases and 5387 controls). In the past 3 years, several studies on this topic have been published and a large number of cases have been accumulated. Furthermore, the genotype and allele analysis was incomplete. In order to get a more precise conclusion of the association between HIF-α C1772T and cancer risk, we conducted a meta-analysis of all eligible case-control studies.

## Methods

### Literature search and data extraction

The methods used for literature search and data extraction has been described elsewhere [41]. In brief, With the term “(cancer or carcinoma) and (Hypoxia-inducible factor-1α or HIF-1α) and (Polymorphism or mutation or variant)”, we found 35 reference in the PubMed, Embase and China National Knowledge Infrastructure (CNKI) database that were published after the first report on HIF-1α(last update: August 23 2013). Selection criteria of an eligible study were (a) investigation of the polymorphisms C1772T of HIF-1α and cancer risk; (b) use of a case-control design based on unrelated individuals and (c) sufficient genotype distributions for cases and controls so that an odds ratio (OR) with 95% confidence interval (CI) could be assessed. If more than one article was published using the same patient population, only the latest or the largest study would be used in our meta-analysis.

The data, including publish year, first author, original country, ethnicity, gender and cancer site (those cancer sites only exist in one article were divided into “other site group”), case number and control number, source of control (population-based or hospital-based) and genotyping method were independently extracted by two investigators and reached conformity on all items through consultation.

### Statistical study

Crude odds ratios (ORs) and 95% confidence intervals (95% CIs) were used to estimate the strength of association between the C1772T polymorphism and cancer risk or lymph node metastasis and they were determined by Z-test. The pooled ORs were performed for the genetic models including dominant model (TT+CT vs.CC), recessive model (TT vs. CT+CC), homozygote comparison (TT vs. CC) and heterozygote comparison (CT vs. CC). The statistical significance of OR was analyzed by Z test, *P* < 0.05 was considered statistically significant. 

In addition to the comparison among all subjects, we also performed stratification analyses by cancer site, ethnicity, source of controls and gender.

Heterogeneity was evaluated by a chi square-based Q statistic, and statistical significance was assumed for *P* value less than 0.05. A fixed-effect model was used when *P* heterogeneity < 0.05, otherwise a random effect model was used. The sensitivity analysis was performed by excluding those studies which did not follow HWE (Hardy-Weinberg equilibrium) to assess the stability of the current analysis [42].

The possible publication bias was examined visually by the Begg’s funnel plot and the degree of asymmetry was tested by Egger’s test.

All of the statistical tests were performed by STATA11.0.

## Results

### Characteristics of studies included

#### Basic characteristics of studies

**Figure 1:**
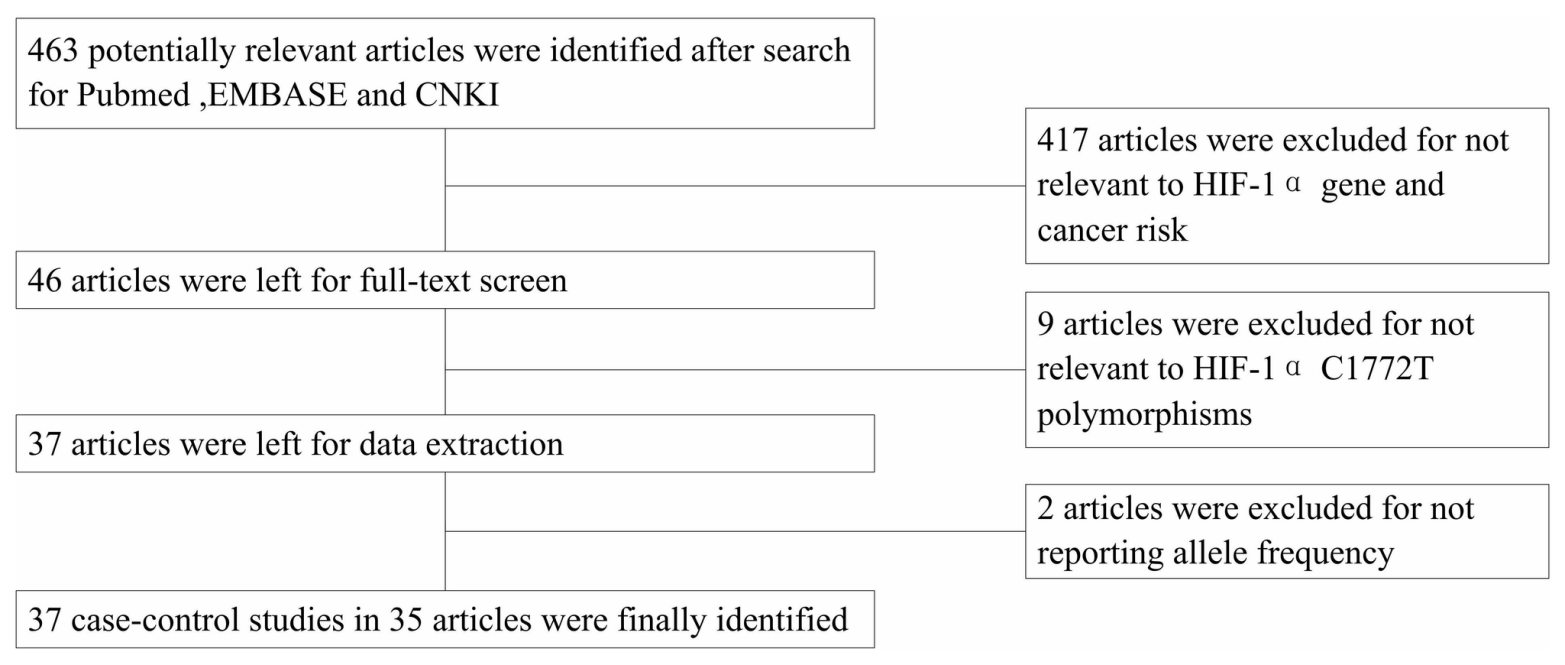
Flow chart for the process of selecting associated publications.

463 relevant publications were identified after initial screening based on our search criteria (up to August 23 2013). Among these, 46 articles were subjected to further examination after reading the titles and abstract. 9 articles were excluded for not relevant to C1772T. 3 articles were excluded for not reporting allele frequency. (Figure **1**) Finally, a total of 37 case-control studies from 35 articles that met the selection criteria (Table **1**) and totally 10186 cases and 10926 controls were used in the pooled analysis. Out of those 37 studies, 6 studies focused on head and neck cancer, 6 on prostate cancer, 6 on breast cancer, 3 on cervical cancer, 3 on lung cancer, 3 on renal cell carcinoma, 2 on colorectal cancer, 2 on pancreatic cancer and one, respectively, on endometrial, gastric, hepatocellular, ovarian cancer, esophageal squamous cell carcinoma and glioma. Among the 35 articles, 1 article provided data of the C1772T polymorphism on three kinds of cancers (cervical cancer, endometrial cancer and ovarian cancer) (Konac et al., 2007）. Then, each type of cancer in the study was treated as a separate study in the meta-analysis. The ethnicities studied were Caucasian (13 articles), Asian (18 articles), mixed population (3 articles) and Brazilian (1 article). The gender studied were female (9 articles), male (6 articles) and mixed (20 articles).

**Table 1:** Characteristics of populations and cancer types of the studies included in the meta-analysis.

Author	Year	Country	Ethnicity	Cancer site	Case-control	Gender**^a^**	Source ofcontrol	Genotypingmethod	HWE
Ribeiro AL	2013	Portugal	Caucasian	Breast cancer	96/74	Female	HB	PCR–RFLP	0
Alves LR	2012	Brazil	Brazilian	Oral squamous cell carcinoma	88/40	Mixed	PB	PCR–RFLP	0
Zagouri F	2012	Greece	Caucasian	Breast cancer	113/124	Female	PB	PCR–RFLP	0.41
Li P	2012	China	Asian	Prostate cancer	662/716	Male	PB	TaqMan	0.27
Mera-Menendez F	2012	Spain	Caucasian	Glottic cancer	121/154	Mixed	PB	PCR–RFLP	0.01
Ruiz-Tovar J	2012	Spain	Caucasian	Pancreatic cancer	59/159	Mixed	PB	PCR-RFLP	0.01
Kuo WH	2012	China	Asian	Non-small-cell lung cancer	285/300	Mixed	PB	PCR–RFLP	0.13
Qin C	2012	China	Asian	Renal cell carcinoma	620/623	Mixed	PB	TaqMan	0.22
Wang X	2011	China	Asian	Pancreatic cancer	263/271	Mixed	PB	PCR-SSCP	0.35
Putra AC	2011	Japan	Asian	Lung cancer	83/110	Mixed	PB	PCR-SSCP	0.55
Kim YH	2011	Korea	Asian	Cervical cancer	199/215	Female	PB	PCR-RFLP	0.32
Xu G	2011	China	Asian	Glioma	150/150	Mixed	HB	PCR-RFLP	0.35
Chai D	2010	China	Asian	Cervical cancer	97/117	Female	HB	PCR-RFLP	0.52
Shieh TM	2010	China	Asian	Oral squamous cell carcinoma	305/96	Mixed	PB	PCR-RFLP	0.71
Hsiao PC	2010	China	Asian	Hepatocellular carcinoma	102/347	Mixed	HB	PCR-RFLP	0.72
Chen MK	2009	China	Asian	Oral cancer	174/347	Mixed	PB	PCR-RFLP	0.72
Naidu R	2009	Malaysia	Asian	Breast cancer	410/275	Female	PB	PCR-RFLP	0.92
Foley R	2009	Ireland	Caucasian	Prostate cancer	95/196	Male	PB	Sequencing	0.62
Konac E	2009	Turkey	Caucasian	Lung Cancer	141/156	Mixed	HB	PCR-RFLP	0.34
Li K	2009	China	Asian	Gastric Cancer	87/106	Mixed	PB	PCR	0.51
Munoz-Guerra MF	2009	Spain	Caucasian	Oral Cancer	74/139	Mixed	PB	PCR-RFLP	0.01
Kim HO	2008	Korea	Asian	Breast cancer	90/102	Female	PB	PCR	0.64
Jacobs EJ	2008	USA	Mixed	Prostate cancer	1425/1453	Male	PB	TaqMan	0.04
Apaydin I	2008	Turkey	Caucasian	Breast Cancer	102/102	Female	PB	PCR-RFLP	0.42
Lee JY	2008	Korea	Asian	Breast cancer	1930/1677	Female	PB	PCR-RFLP	0.25
Li H	2007	USA	Mixed	Prostate Cancer	1072/1271	Male	PB	PCR-RFLP	0.16
Konac E-O	2007	Turkey	Caucasian	Ovarian	49/107	Female	PB	PCR-RFLP	0.23
Konac E-C	2007	Turkey	Caucasian	Cervical	32/107	Female	PB	PCR-RFLP	0.23
Konac E-E	2007	Turkey	Caucasian	Endometrial	21/107	Female	PB	PCR-RFLP	0.23
Orr-Urtreger A	2007	Israel	Caucasian	Prostate Cancer	402/300	Male	PB	PCR-SSCP	0.14
Fransen K	2006	Sweden	Caucasian	Colorectal Cancer	198/258	Mixed	PB	PCR-RFLP	0.92
Ling TS	2005	China	Asian	Esophageal squamous cell carcinoma	95/104	Mixed	HB	PCR-RFLP	0.57
Chau CH	2005	USA	Mixed	Prostate cancer	196/196	Male	PB	Sequencing	0.01
Kuwai T	2004	Japan	Asian	Colorectal cancer	100/100	Mixed	PB	PCR-SSCP	0.56
Ollerenshaw M	2004	UK	Caucasian	Renal cell carcinoma	160/288	Mixed	PB	PCR-SSCP	0
Tanimoto K	2003	Japan	Asian	Head and neck squamous cell carcinoma	55/110	Mixed	PB	Sequencing	0.55
Clifford SC	2001	UK	Caucasian	Renal cell carcinoma	35/143	Mixed	HB	PCR-SSCP	0.02

^a^ Mixed means samples contain both female and male;

PB, public based; HB, hospital based; HWE, Hardy-Weinberg equilibrium; PCR, polymerase chain reaction; RFLP, restriction fragment length polymorphism; TaqMan, TaqMan SNP Genotyping Assays.

#### Characteristics of allele average frequency

**Figure 2:**
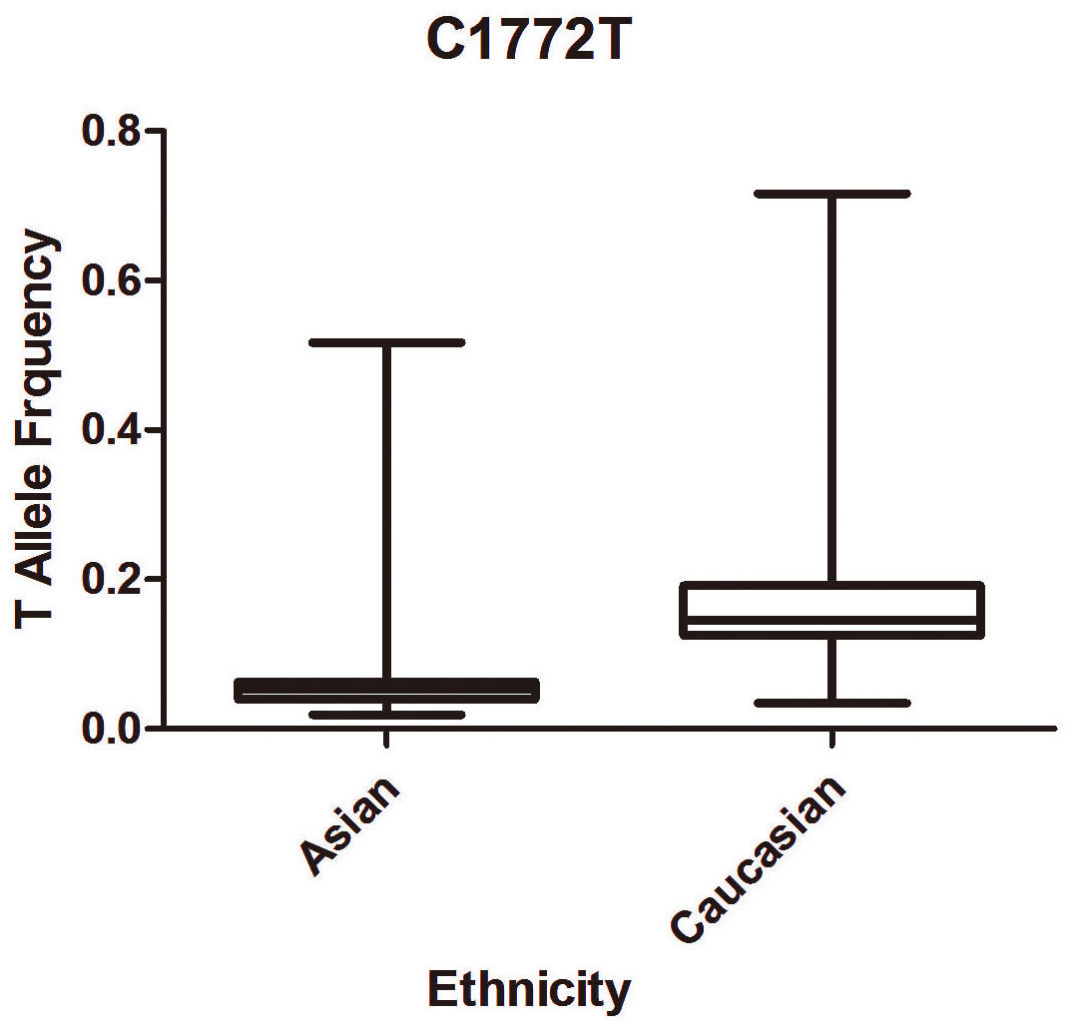
T allele frequency of HIF-1α C1772T among control subjects stratified by ethnicity.

In the control group, T allele average frequency of HIF-1α C1772T among Asian populations (0.06) were lower than Caucasian populations (0.18) and it has statistical significance for *P*=0.004. (Figure **2**)

### Meta-analysis

**Figure 3:**
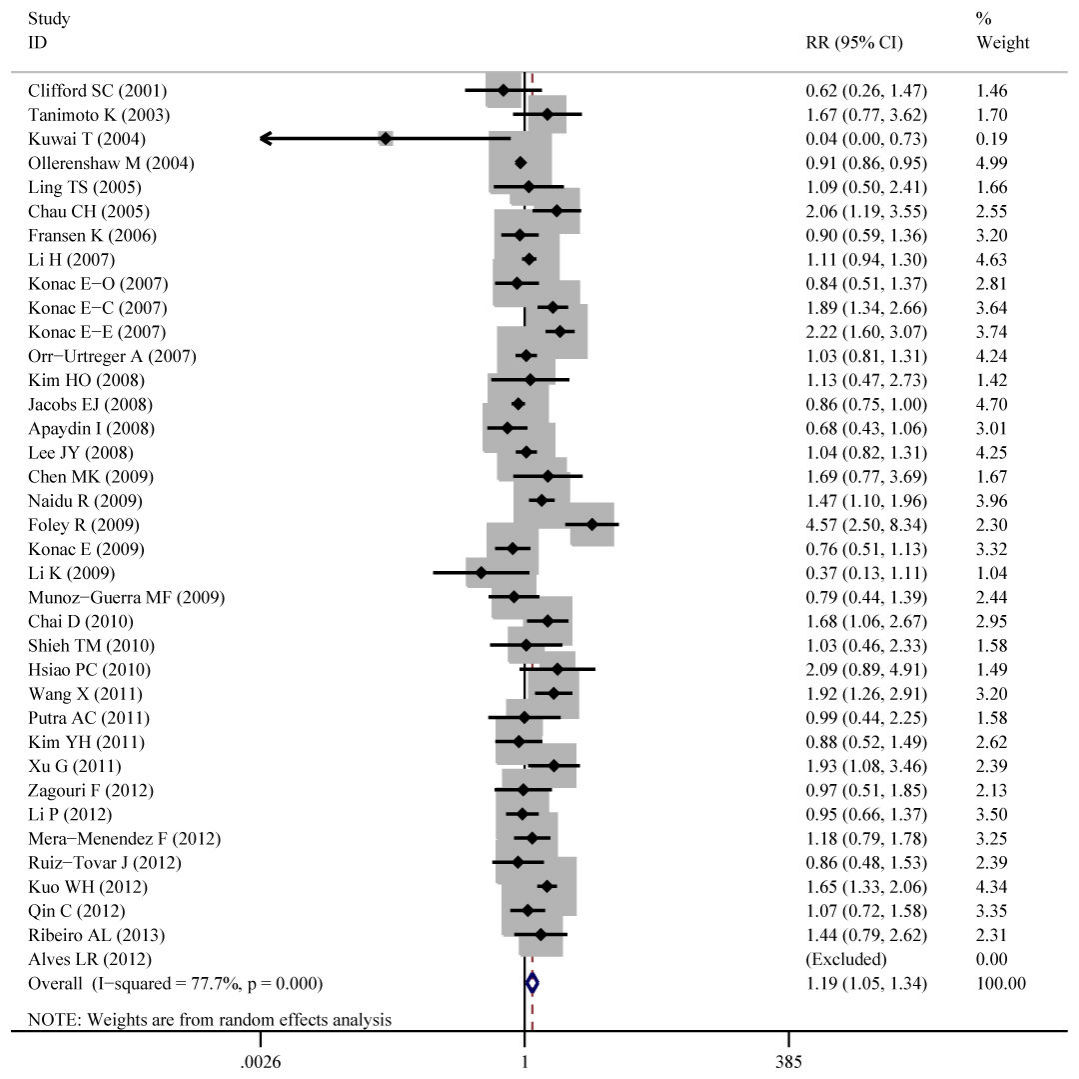
Forest plot of cancer risk associated with C1772T (TT +CT vs. CC). The squares and horizontal lines correspond to the OR and 95%CI. The area of the squares indicates the study-specific weight.

Aggregated ORs and heterogeneity test result for the association between C1772T and cancer risk was shown in Table **2** and the result of TT+CT vs.CC (dominant model) was shown in Figure **3** **-**Figure **7**.

**Table 2:** Associations between the C1772T polymorphism and cancer risk.

Variables	TT+CT VS CC			TT VS CT +CC			TT VS CC			CT VS CC		
	**n^a^**	OR(95%CI)	*P* ^b^	**n^a^**	OR(95%CI)	*P* ^b^	**n^a^**	OR(95%CI)	*P* ^b^	**n^a^**	OR(95%CI)	*P* ^b^
Overall	36	**1.23(1.03-1.47)**	0	26	**2.51(1.54-4.09)**	0	25	**2.02(1.21-3.39)**	0	36	1.16(0.97-1.38)	0
Overall for HWE**^d^**	28	**1.31(1.08-1.60)**	0	17	**2.77(1.59-4.82)**	0.01	17	**3.04(1.62-5.69)**	0	28	**1.24(1.03-1.49)**	0
cancer cite												
Breast cancer	6	1.12(0.87-1.52)	0	5	1.64(0.56-4.77)	0.11	5	1.69(0.56-5.14)	0.09	6	1.10(0.83-1.46) **^c^**	0.16
Cervical cancer	3	1.81(0.79-4.10)	0.01	2	**8.80(2.31-33.52) ^c^**	0.24	2	**11.49(2.21-59.67) ^c^**	0.16	3	1.47(0.79-2.74) **^c^**	0.29
Colorectal cancer	2	0.26(0.01-5.09)	0.03	1	1.97(0.33-11.90)	0	1	1.91(0.32-11.58)	0	2	0.25(0.01-4.69)	0
Head and neck cancer	5	1.20(0.87-1.67) **^c^**	0.48	4	**11.29(1.24-103.02) ^c^**	0.79	3	**2.24(1.14-4.39) ^c^**	0.72	5	1.03(0.69-1.62) **^c^**	0.92
Lung cancer	3	1.19(0.51-2.76)	0	2	1.39(0.09-21.85)	0.07	2	1.42(0.07-29.73) **^c^**	0.05	3	1.13(0.59-2.19) **^c^**	0.16
Pancreatic cancer	2	1.39(0.54 -3.56)	0.03	1	**4.13(1.57-10.86)**	0	1	**3.39(1.28-8.97)**	0	2	0.51(0.02-11.53)	0
Prostate cancer	6	1.36(0.95-1.96)	0	5	1.31(0.54-3.18)	0.01	5	1.34(0.54-3.30)	0.01	6	1.34(0.93-1.92)	0.03
Renal cell carcinoma	3	0.46(0.13-1.60)	0.01	3	**1.55(1.02-2.37) ^c^**	0.47	3	0.29(0.06-1.45) **^c^**	0.21	3	0.44(0.11-1.69) **^c^**	0.42
Other cancer	6	1.46(0.72-2.96)	0	3	3.98(0.69-22.67)	0.14	3	5.04(0.47-54.13)	0.04	6	1.39(0.72-2.68) **^c^**	0.33
Subgroup by ethnicity												
Caucasian	15	1.13(0.78-1.65)	0	13	**2.20(1.28-3.78)**	0.01	13	1.78(0.80-3.94) **^c^**	0.95	15	0.98(0.65-1.48)	0.01
Asian	18	**1.34(1.08-1.67)**	0	9	**3.71(2.26-6.08) ^c^**	0.97	9	**4.20(2.55-6.92) ^c^**	0.95	18	**1.27(1.04-1.56**)	0.01
control source												
PB	29	1.21(0.99-1.47)	0	21	**2.92(1.71-4.99)**	0	20	**2.26(1.27-4.03)**	0	29	1.12(0.92-1.36)	0.01
HB	7	1.33(0.87- 2.03)	0.03	5	1.15(0.39-3.34) **^c^**	0.26	5	1.21(0.39-3.74) **^c^**	0.21	7	1.35(0.91-2.00) **^c^**	0.08
Gender												
Female	11	1.34(0.97-1.86)	0	9	**3.17(1.27-7.91)**	0.01	9	**3.72(1.29-10.74)**	0	11	1.23(0.93-1.62)	0.02
Male	6	1.36(0.95-1.96)	0	5	1.31(0.54-3.18)	0.01	5	1.34(0.54-3.30)	0.01	6	1.34(0.93-1.92)	0.03

^a^ Number of comparisons.

^b^ *P* value of Q-test for heterogeneity test.

^c^ Fixed-effects model was used when *P* value for heterogeneity test≥0.05; Otherwise, Random-effects model was used.

^d^ data after excluding those studies’ controls not in Hardy-Weinberg equilibrium.

**Figure 4:**
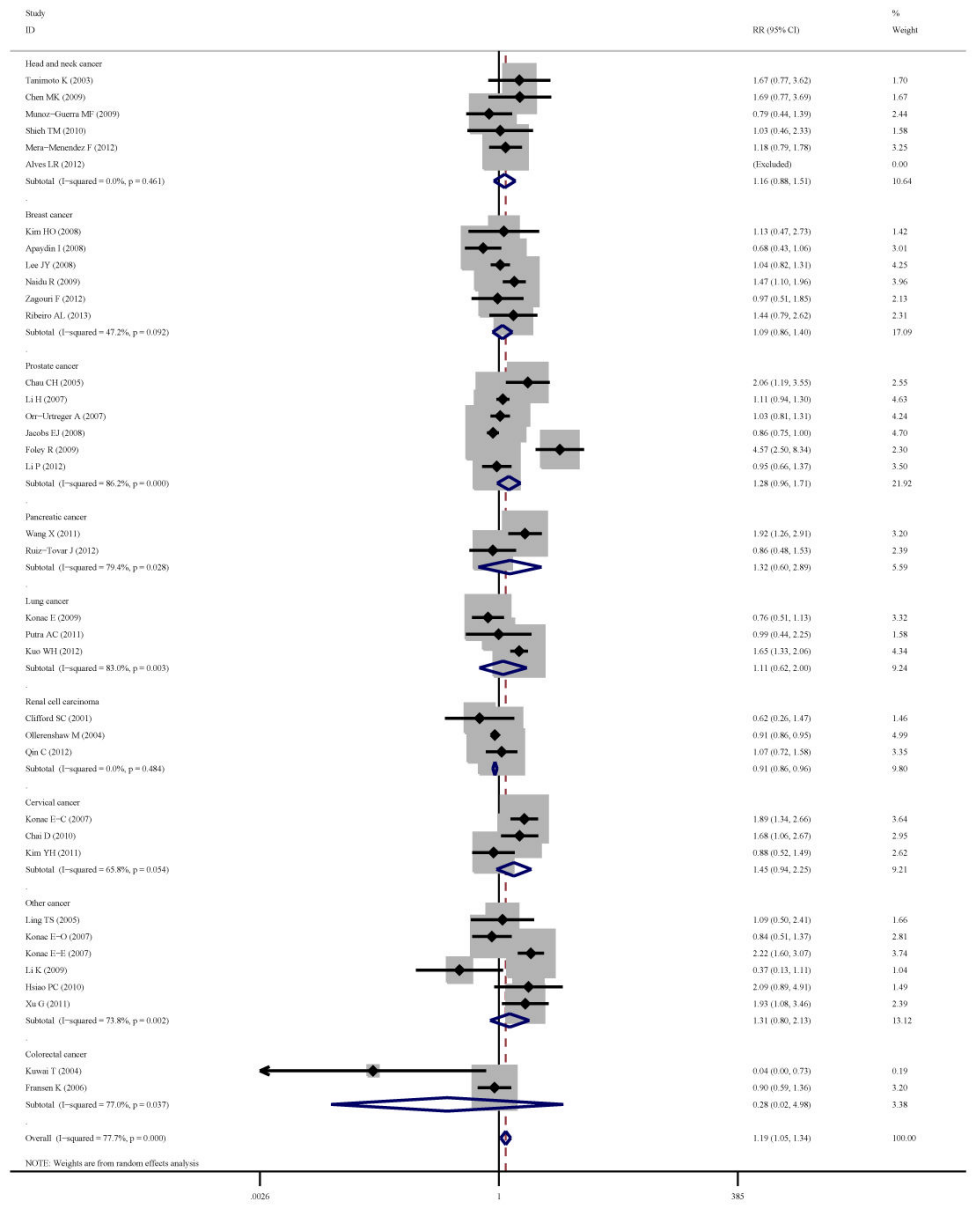
Forest plot of cancer risk associated with C1772T (TT +CT vs. CC) stratified by cancer site.

**Figure 5:**
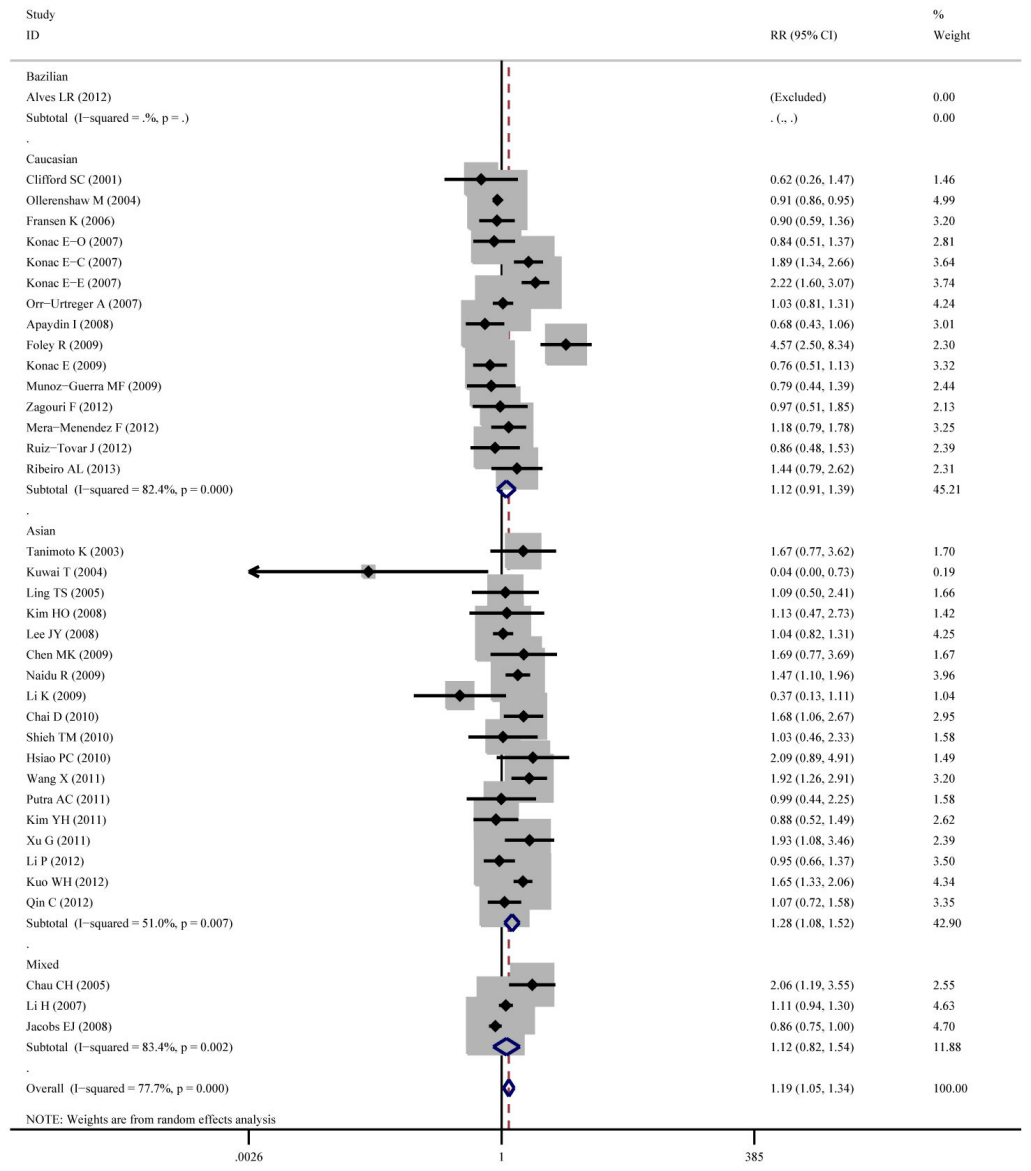
Forest plot of cancer risk associated with C1772T (TT +CT vs. CC) stratified by ethnicity.

**Figure 6:**
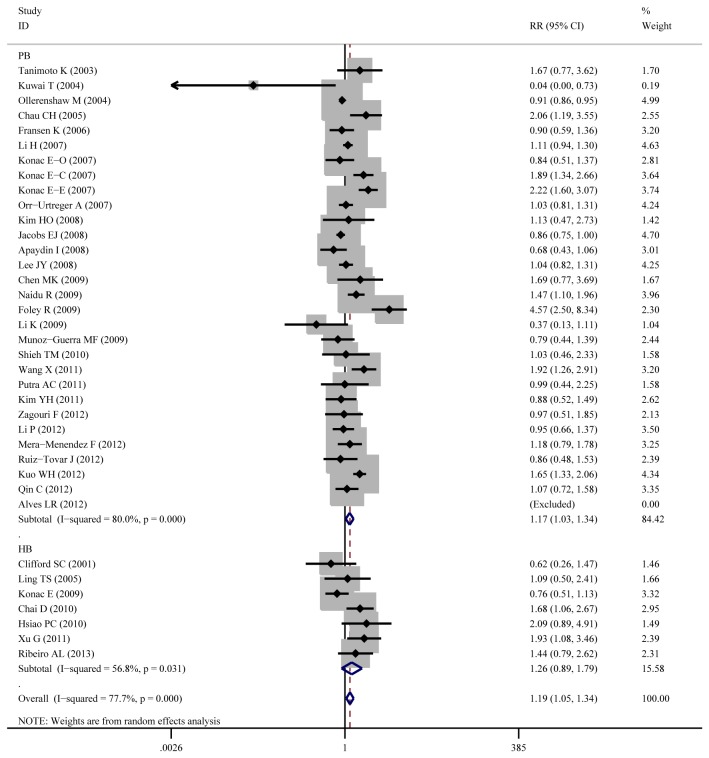
Forest plot of cancer risk associated with C1772T (TT +CT vs. CC) stratified by source of control.

**Figure 7:**
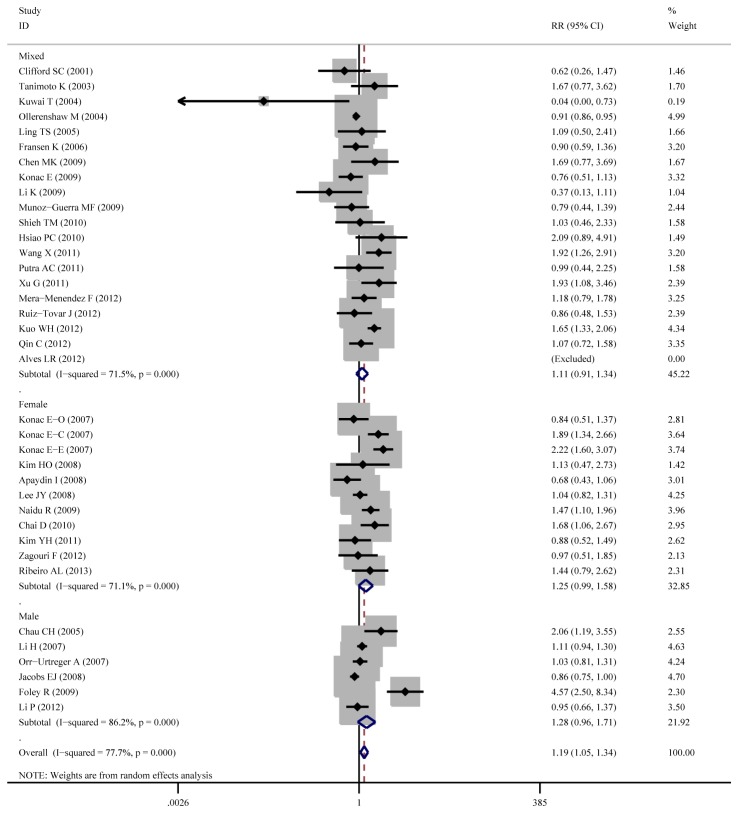
Forest plot of cancer risk associated with C1772T (TT +CT vs. CC) stratified by gender.

Overall, the C1772T was found to be significantly associated with increased cancer risk for three genetic models: TT+CT vs.CC (dominant model OR=1.23, 95%CI=1.03-1.47), TT vs. CT+CC (recessive model OR=2.51, 95%CI=1.54-4.09), TT vs. CC (homozygote comparison OR=2.02, 95%CI=1.21-3.39).

As for cancer site subgroup, significant association exists in the following four cancer sites: Cervical cancer, for TT vs. CT+CC (OR=8.80, 95%CI=2.31-33.52) and TT vs. CC (OR=11.49, 95%CI=2.21-59.67). Pancreatic cancer, for TT vs. CT+CC (OR= 4.13, 95%CI= 1.57-10.86) and TT vs. CC (OR= 3.39, 95%CI= 1.28-8.97). Head and neck cancer, for TT vs. CT+CC (OR= 11.29, 95%CI= 1.24-103.02) and TT vs. CC (OR= 2.24, 95%CI= 1.14-4.39). Renal cell carcinoma for TT VS CT +CC (OR=1.55, 95%CI=1.02-2.37).

As for ethnicity subgroup, significant association exists among Asian population for TT+CT vs.CC (OR=1.34, 95%CI=1.08-1.67), TT vs. CT+CC (OR=3.71, 95%CI=2.26-6.08), TT vs. CC (OR=4.20, 95%CI=2.55-6.92) and CT vs. CC (OR=1.27, 95%CI=1.04-1.56). However, in Caucasian subgroup, significant increased risk only exist for TT vs. CT+CC (OR=2.20, 95%CI=1.28-3.78) and not for other genetic models.

As for gender subgroup, significant association exists in female group for TT vs. CT+CC (OR=3.17, 95%CI=1.27-7.91) and TT vs. CC (OR=3.72, 95%CI=1.29-10.74).

### Heterogeneity analysis and Sensitivity analysis

Significant heterogeneity was found in overall comparisons under all four genetic models (dominant model *P*<0.01, recessive model *P*<0.01, homozygote comparison *P*<0.01 and heterozygote comparison *P*<0.01). 

As for eight studies included in our meta-analysis which did follow HWE (Table **1**), we performed the sensitivity analysis by excluding those studies to assess the stability of the current analysis. All the obtained results were similar except for CT vs. CC genetic model (OR=1.24, 95%CI=1.03-1.49). (Table **2**)

### Publication bias

**Figure 8:**
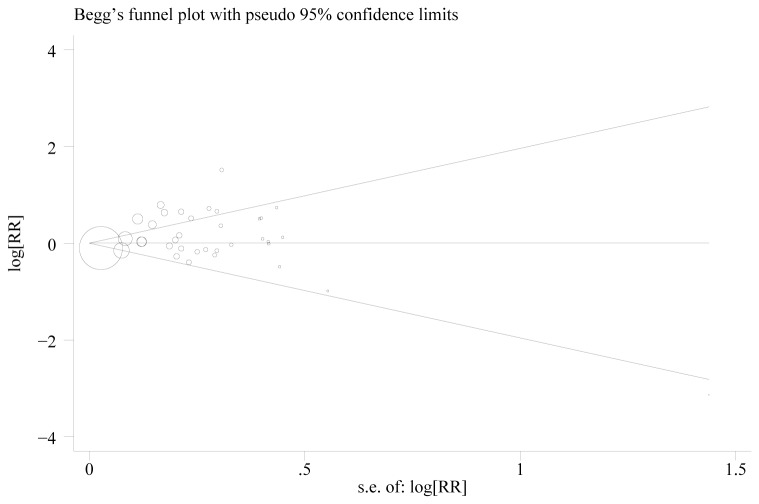
Funnel Plot for Publication Bias of the C1772T Polymorphism. Each point represents a separate study for the indicated association. Log (OR): natural logarithm of OR. Horizontal line: mean effect size.

The shape of the funnel plots did not reveal any evidence of obvious asymmetry (Figure **8**), suggesting that there was no obvious publication bias. Egger’s test was used to provide further statistical evidence; similarly, the results showed no significant publication bias in this meta-analysis (t =0.69, *P* = 0.50 for TT+CT vs. CC).

## Discussion

HIF-1 is a key transcription factor that regulates cellular reaction to hypoxia and is over expressed in most solid tumors in response to low oxygen concentrations [3]. It has the ability to influence metabolic reprogramming, angiogenesis, metastasis and recent studies found that HIF-1α was associated with many kinds of cancer [3,6,7]. 

HIF-1 gene polymorphisms have been investigated for a possible role in mediating genetic predisposition to cancer[34,43,44] and C1772T is one of important SNP of human HIF-1α gene [3]. The possible association between C1772T and cancer risk has been studied by several investigators, but the results were inconsistent. Meta-analysis is a powerful tool for summarizing the results from different studies which provide more reliable results than a single case-control study [34]. 

In this meta-analysis we investigated the association between C1772T and cancer risk. Totally 10186 cases and 10926 controls were included and subgroup analysis by cancer site, ethnicity, source of controls and gender was performed. 

In overall meta-analysis, the T allele of C1772T was found to be significantly associated with an increased cancer risk in all four genetic models. The T allele of C1772T may influence the development of cancer through the following mechanisms. First, it may increase the transactivation capacity of HIF-1α. As a result, HIF-1α-regulated genes expression increased, leading to enhanced angiogenesis [3]. Also, Fu et al found that T allele of C1772T may enhance the HIF-1α stability that increases tumor susceptibility [45].

As for the subgroup analysis by cancer site, the T allele of C1772T influence outcome varied in different cancer sites. Obvious association between T allele of C1772T and increased cancer risk exists in cervical cancer, pancreatic cancer and head and neck cancer, while not associated with colorectal cancer, lung cancer, breast cancer, prostate cancer and other cancers. Cancer of different sites has variant tumor microenvironment that regulates or influences the gene expression profiles. The same polymorphism may exert different effects in variant cancers [46,47]. However, all those results should be treated with reservation as there were only 2 or 3 studies included for some cancer site subgroup which may reduce the results’ reliability.

In the subgroup of ethnicity, significantly increased cancer risks with T allele of C1772T were found in Asian population under all four genetic models. As for Caucasian subgroup, significant increased risk only exists under recessive model. Possible explanations were as follows, First, genetic background varies among different ethnicities [48], as listed in the result part, the T allele of C1772T between Asian and Caucasian was statistically different (*P*=0.004). Second, the outer environment and life-styles were different between Asian and Caucasian which may influence the causal and development of cancer. Third, relatively deficient studies and sample size may limit the districts and kinds of ethnicities in our analysis and could enhance the bias in our analysis.

As for subgroup of gender, we found significantly increased cancer risk with T allele of C1772T in female subgroup instead of male subgroup. This may be explained by the following reasons. First, estrogen and progestin can enhance the expression of HIF-1α via Akt signaling pathway which could cause cancer cell angiogenesis or invasion [49,50]. Second, only female-specific cancer (breast cancer, ovarian cancer, cervical cancer and endometrial cancer) were included in female subgroup, while no data reported non-female-specific cancer such as lung cancer. Third, female subgroup contains 5932 samples, while totally 21112 samples were included in this meta-analysis. As a result, the overall results may be influenced by gender difference. Therefore, more studies on C1772T and non-gender-specific cancer risk among different genders are called for in the future.

In this study, obvious heterogeneity was found in all four genetic models under both overall and subgroup analysis. Totally eight studies included in our meta-analysis did not follow HWE. Deviation from HWE reflects potential mistakes existing in those eight studies, such as laboratory or genotyping errors, population stratification or selection bias in the choice of controls [42,51]. The sensitivity analysis was performed by excluding those 9 studies deviated from HWE to assess the stability of the current analysis. All the obtained results were similar except for the CT vs. CC genetic model. The varying result of association between C1772T heterozygote and cancer risk calls for more related case-control studies.

The current meta-analysis has several limitations which should be noted. First, although 34 studies and totally 21112 samples were included in our study, the sample size for some subgroup analysis was limited which could increase the likelihood of type I and type II errors. Second, cancer is the result of the influence of genetic and environmental factors and the latter was not analyzed in this study. Therefore, it is necessary to assess the roles of some environment factors such as diet, life style, tobacco and so on. Third, publication bias was not avoidable although our funnel plot and Egger’s test did not show any bias, as for positive results were much more likely to be published. 

Despite those limitations above, our meta-analysis also has some advantages. First, it contains the latest data about association between C1772T of HI F-1α gene polymorphism and cancer risk. Second, we conducted four types of genotype analysis and subgroup analysis by cancer site, ethnicity, source of controls and gender.

## Conclusions

Our meta-analysis suggests that the substitution of C to T of HIF-1α gene C1772T polymorphism is a risk factor of cancer, especially for cervical, head and neck cancer, pancreatic cancer and renal cell carcinoma. Also, a significant increase in cancer was observed in Asian group as well as in female group.

## Supporting Information

Figure S1 **Preferred reporting items for systemic reviews and meta-analysis (PRISMA) flow chart.**
(DOC)

Checklist S1 **PRISMA check list.**
(DOC)
