# Supplementary material for: The Association between Hypoxia-Inducible Factor-1 α Gene C1772T Polymorphism and Cancer Risk: A Meta-Analysis of 37 Case-Control Studies
Source: PLoS One. 2013 Dec 18;8(12):e83441. doi: 10.1371/journal.pone.0083441 (PMC3867430; doi:10.1371/journal.pone.0083441)
Supplement: Figure S1 — Preferred reporting items for systemic reviews and meta-analysis (PRISMA) flow chart. (DOC) [file pone.0083441.s001.doc]

**Identification**

**Screening**

**Eligibility**

**Included**

463 of records identified through database searching

0 of additional records identified through other sources

0 of records after duplicates removed

46 of records screened

417 of records excluded

37 of full-text articles assessed for eligibility

37 of articles included in qualitative synthesis

9 of full-text articles excluded, for not relevant to HIF1-ΑC1772T polymorphism

37case-control studies in 35 articles included in quantitative synthesis (meta-analysis)
